# The suppression of ocean waves by biogenic slicks

**DOI:** 10.1098/rsif.2024.0385

**Published:** 2024-11-13

**Authors:** Nathan J. M. Laxague, Christopher J. Zappa, Shantanu Soumya, Oliver Wurl

**Affiliations:** ^1^ University of New Hampshire, Durham, NH, USA; ^2^ Lamont-Doherty Earth Observatory of Columbia University, Palisades, NY, USA; ^3^ Carl von Ossietzky Universitat Oldenburg, Oldenburg, DE, Germany

**Keywords:** waves, air–sea interaction, surfactants, wave damping, rheology

## Abstract

Ocean waves are significantly damped by biogenic surfactants, which accumulate at the sea surface in every ocean basin. The growth, development, and breaking of short wind-driven surface waves are key mediators of the air–sea exchange of momentum, heat and trace gases. The mechanisms through which surfactants suppress waves have been studied in great detail through careful laboratory experimentation in quasi-one-dimensional wave tanks. However, the spatial scales over which this damping occurs in structurally complex surfactant slicks on the real ocean have not been resolved. Here, we present the results of field observations of the spatial response of decimetre- to millimetre-scale waves to biogenic surfactant slicks. We found that wave damping in organic material-rich coastal waters resulted in a net (spatio-temporally averaged) reduction of approximately 50% in wave slope variance relative to the open ocean for low to moderate wind speeds. This reduction of wave slope variance is understood to result in a corresponding reduction in momentum input to the wave field. This significant effect had thus far evaded quantification due in large part to the enormous range of scales required for its description—spanning the sea surface microlayer to the ocean submesoscale.

## 1. Introduction

**Figure 1.**
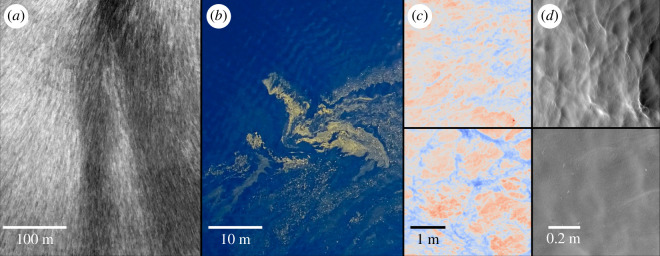
Visualizations of biogenic sea slicks over a range of scales. (*a*) backscattered power from X-band marine radar, (*b*) airborne colour image featuring a prominent bloom of *Trichodesmium* cyanobacteria, (*c*) longwave infrared skin temperature field within clean (top) and slick (bottom) regions, (*d*) corresponding surface wave slope fields. All panels except (*b*) were produced from data collected from the same region at very nearly the same time. Photo in (*b*) was taken later/elsewhere and appears courtesy of Alex Ingle & the Schmidt Ocean Institute.

The ocean surface layer’s uppermost hundreds of micrometres—the sea surface microlayer (SSML)—is distinct from underlying ocean water in its biological [1], chemical [2] and physical [3] properties. In the tropics, where low winds and warm waters prevail, this sublayer is often rich in microorganisms [2,3] capable of producing substances that modify the physical properties of the surface ocean water. These surface-active substances (SAS), often referred to as surfactants, are organic compounds that accumulate at the air-side molecular layer above the air–water interface, a direct consequence of the orientation and water-repellent effect of the hydrophobic groups of surfactants accumulated in the SSML [4]. The presence of these compounds is strongly associated with the suppression of water waves [5] and occurs through a number of mechanisms: (i) the reduction of the air–water surface tension [6], the restoring force for capillary waves, (ii) the enhancement of seawater’s elastic modulus [7,8], increasing viscous damping, and (iii) the generation of longitudinal Marangoni waves [9–11], which come into resonance with and therefore attenuate transverse surface gravity waves, a phenomenon known as ‘Marangoni–Gibbs damping’. Centimetre- to metre-length ocean waves carry the majority of wave-supported stress and play an essential role in defining the surface roughness, which mediates the flux of momentum into longer waves. The suppression of these waves significantly reduces wind input into the wave field at all scales (waveform stress) [9,12] and the dissipation of waves due to breaking [13,14]. The reduction of waveform stress results in the enhancement of wind speed over surface slicks [15,16], while the calming of surface wave breaking increases the characteristic length scale of surface temperature features [17,18] (figure 1*c*) and reduces the air–sea gas transfer velocity [19]. At high levels of surfactant concentration, the lifetime of bubbles increases, resulting in the formation of persistent bubble rafts [20].

Surfactants tend to accumulate along regions of ocean surface water convergence—e.g. ocean fronts or Langmuir circulation (figure 1*a*). However, slicks and the microorganisms that create them are readily stretched and deformed by smaller-scale turbulent eddies, producing meandering streaks that intermittently break and reattach under varying environmental conditions (figure 1*b*). The heterogeneous distribution of surfactant results in spatial variation in surface temperature [3,18] (as in figure 1*c*) and surface roughness (as in figure 1*d*) that evolves over scales ranging from decimetres to hundreds of metres and larger. Investigation of spatially varying wave damping characteristics requires high-resolution measurements of realistic surface waves and chemistry over a large domain, limiting such study to the real ocean. In the past, damping has been quantified via microwave radar remote sensing [10], selective sampling via capacitance gauge [21], or novel instrumentation on remotely controlled platforms [19,22]. Even so, the available body of field observations of surface wave damping is sparse, and almost exclusively limited to the wave damping ratio—the ratio of the undamped wave spectrum to the damped wave spectrum. However, knowledge of the spatial variation of the surface wave field gives us an even greater insight into the modification of air–sea interaction by chemical surfactants. One of the key measures of this spatial variation is the damping coefficient, the inverse length scale of exponential decay for wave amplitudes in the presence of slicks [23].

Biogenic changes to SSML chemistry have a remarkable impact on air–sea interaction. Turbulent fluxes of momentum, heat and gas are strongly affected by the sea surface temperature (SST) and surface roughness. When making direct measurements of these fluxes, it is variation within the (approx. 100–1000 m) upstream flux footprint that is of greatest importance to the exchange [24]. Due to the challenges associated with directly measuring turbulent air–sea fluxes, they are typically parametrized in terms of scalar environmental state variables such as average values of wind speed, air pressure, relative humidity and the air–sea temperature difference [25]. However, at low to moderate levels of wind forcing, microscale wave breaking is the physical process which regulates air–sea fluxes of heat and gas [26]. The presence of SAS affects the dynamics of wave breaking [14,27] to the degree that these fluxes are inhibited [26,28,29].

**Figure 2.**
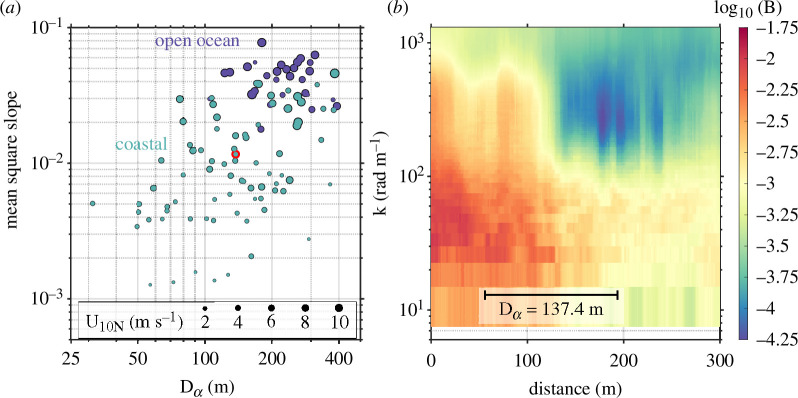
(*a*) Variation of wave slope variance (mean square slope) with damping length scale $D_{\alpha }$. (*b*) Wavenumber saturation spectrogram computed along 100 m of the ship transit path. Damping length scale ($D_{\alpha }$ = 137.4 m) for this particular run is outlined in red on panel (*a*).

Here, we present results from field observations of surface wave damping in the presence of spatially heterogeneous biogenic slicks in coastal and open ocean waters of the tropical Pacific. We resolved the spatial variation of the surface wave field for wavelengths of order of 0.001–1 m over lateral length scales of 1 km, allowing us to determine the wavenumber-dependent damping ratio and damping coefficient. In tandem with the wave measurements, we simultaneously measured key chemical properties of the SSML (including concentration of SAS, chromophoric dissolved organic matter (CDOM) and chlorophyll-A) via remotely operated catamaran. We found a persistent regional distinction: surface waves were weakly damped in open ocean waters, while significant damping occurred over short spatial scales in coastal waters (figure 2*a*). This distinction is owed not only to the spatially averaged concentration of surfactants over a given region, but also to the spatial damping characteristics of individual slick filaments (figure 2*b*).

## 2. Methodology

### 2.1. Observational tools

**Figure 3.**
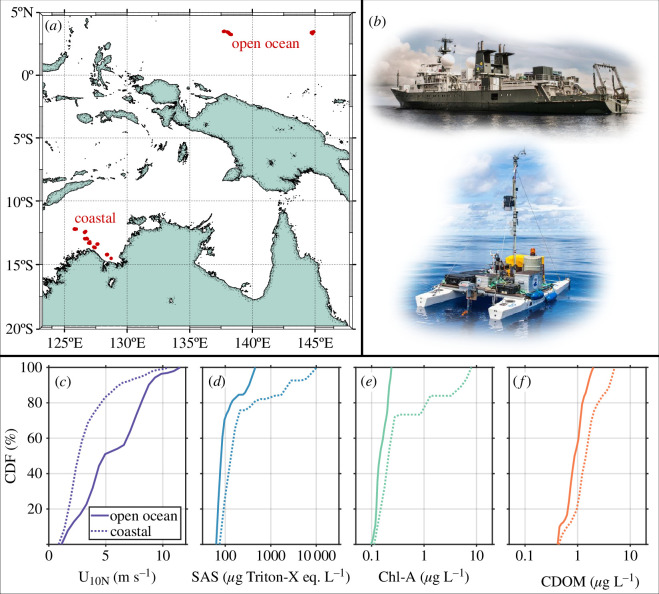
(*a*) Map of the East Timor Sea and Western Equatorial Pacific, with locations of intensive measurement marked by red dots, (*b*) *R/V Falkor* and S${,}^{3}$ catamaran, (*c,f*) cumulative density functions (CDFs) for the 10 m wind speed given neutral stability ($U_{10N}$) and SSML concentrations of surface-active substances (SAS), chlorophyll-A (Chla-A) and chromophoric dissolved organic matter (CDOM). Solid and dotted traces indicate CDFs for open ocean and coastal data, respectively. Photos in (*b*) courtesy of Alex Ingle & Schmidt Ocean Institute.

For two months in autumn of 2016, the *R/V Falkor* and a remotely controlled catamaran (figure 3*b*) served as air–sea interaction observational platforms in the East Timor Sea and Western Equatorial Pacific Ocean. The ship was outfitted with state-of-the-art polarimetric and infrared imaging systems, while the catamaran was used to measure surface chemical properties (e.g. SAS, dissolved organic matter and chlorophyll concentrations). This grouping—in concert with the ship’s meteorological package—allowed for the recovery of parameters relevant to atmospheric forcing, wave properties and the chemical and physical processes that impact them (figure 3*c*–*f*).

#### 2.1.1. Shipboard imaging systems

Polarimetric and infrared cameras were mounted on the railing of the 02 deck of the *R/V Falkor* and oriented forward and away of the starboard bow [3,30]. The instantaneous attitude of each camera was logged by a corresponding strapped-down IMU (MicroStrain and Xsens). These nine degree-of-freedom IMUs (three-dimensional accelerations, rotation rates and magnetometre readings) yielded the Euler angles after processing via Kalman filter. Each camera frame was rectified according to the instantaneous camera attitude and position [31,32]. The polarimetric camera enabled near-field remote sensing of the ocean surface wave slope field via polarimetric slope sensing (PSS) [33]. In the PSS technique, the polarization state of light reflected from the sea surface is used to infer the two-dimensional slope of the sea surface at each pixel. The instrument used for the field observations described here was a custom-built Polaris Ursa polarimeter [34], which allowed for high spatial (approx. 1 mm) and temporal (approx. 0.1 s) resolution of the wave slope field over a patch of water of nominal size 1 × 1 m. The infrared imager (a Sofradir MiTIE-cooled long-wave infrared (LWIR) camera with noise equivalent temperature difference (NETD) less than 20 mK) was used to observe the spatio-temporal variation in the ocean skin temperature field. Over the course of three weeks, we acquired 217 individual 20 min segments of imager data (from both polarimetric and infrared cameras) as the ship transited slowly through regions of interest. In order to outrun the hull reflections for the wave scales, which we resolved in our 1 × 1 m field of view and, to ensure that the imaged surface was uncontaminated by industrial surfactants originating from the ship, the vessel targeted a speed of 0.5 m s^−1^ over ground, varying between 0.3 and 1 m s^−1^ as other considerations necessitated. Of our 217 individual acquisitions, 142 were determined to be free of contamination by sun glint. These quality control-passed cases were split across 82 in coastal waters and 60 in the open ocean (figure 3*a*).

#### 2.1.2. S^3^ catamaran

Sea-Surface Scanner (S^3^) [35] is a remotely controlled catamaran with an integrated rotating glass disc assembly and flow-through sensors for high-resolution mapping of the skin layer. The partially immersed glass discs rotate through the SSML, pick it up due to the physical phenomena of surface tension and are scraped off by a set of wipers placed between the discs [36]. The collected SSML and in parallel collected bulk water (1 m) are pumped through temperature and conductivity sensor, and a fluorimeter to measure fluorescent dissolved organic matter (FDOM). The collected water, both SSML and bulk water, can be redirected to a bottle carousel to collect multiple discrete water samples via remote command by the pilot. Dissolved surfactants were analysed by phase-sensitive alternating current voltammetry with a hanging mercury drop electrode according to a technique validated in a European intercomparison study [37]. Throughout the field operations described here, the S^3^ measurements were coordinated with the ship-based observations in order to ensure that the waters sampled by S^3^ were analogous to those sensed by the polarimeter. The measurements of SSML chemistry (e.g. SAS) were obtained over 5–30 min periods, precluding the correlation of particular features within a given 20 min camera acquisition.

#### 2.1.3. Sensing of mean environmental conditions

The *R/V Falkor* possessed onboard environmental sensing capabilities, which we used to characterize the ambient meteorological conditions. The wind speed/direction, air temperature, relative humidity and air pressure were all measured by a Gill MetPak on the foremast (12 m above the mean water level). The water temperature was taken from a Seabird SBE-X mounted at the ship intake, 3 m below the mean water level. These data were passed into the COARE 3.0 algorithm [38] in order to obtain a bulk parametrization of the 10 m wind speed in neutral conditions, $U_{10N}$. The long surface gravity wave field was sensed via the ship’s X-band marine radar antenna, a Raytheon MkII, which sampled at 34.29 MHz with a 2.78 s repetition time. Backscattered power was averaged in time to produce 4096 azimuthal bins and 874 range bins per revolution. When georeferenced and averaged over many minutes of acquisition, these data provided visualization of surface slick patterns (figure 1*a* and figure 8*b*). The individual scans are useful in their own right: the Rutter *Sigma* S6 WaMoS II, real-time processing software utilizes a modulation transfer function to convert the backscattered power into the water surface vertical displacement field [39]. For every 32 antenna rotations, the Earth-referenced wavenumber-frequency directional spectrum was computed, and the wavenumber directional spectrum was saved to file.

### 2.2. Processing techniques

#### 2.2.1. Computation of wave spectra

**Figure 4.**
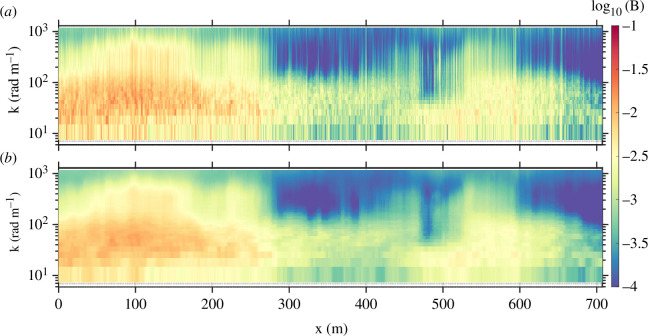
(*a*) Omnidirectional wave saturation spectrogram, with non-overlapping 1 s FFT windows. (*b*) Same spectrogram, subjected to 21 s temporal moving median filter.

The surface slope fields were subjected to Tukey (tapered cosine) windows in order to mitigate the impact of spurious low-wavenumber effects in the spectra [31]. There were two principal constraints in mind with respect to the fast Fourier transform (FFT) window length. On the one hand, the window needed to be long enough for the computed spectral energy density estimates to be robust. On the other hand, the window needed to be short enough to provide resolution of temporal (and therefore spatial) variability in the wave field. A window length of one second was used in order to resolve the variation of short-wave characteristics with respect to the phase of the dominant 5–10 s surface gravity waves [32]. For our purposes here, a window length far shorter than the dominant wave period allowed us to avoid aliasing the hydrodynamic modulation by gravity waves into lower frequencies (and therefore misinterpreting that modulation as wave damping). This surface gravity wave signature was then minimized by subjecting the full spectrograms to a 21 s moving median filter (figure 4).

This spectrogram is not presented in terms of time t; rather, it is presented in terms of ship displacement X. This quantity was computed through processing of the persistent and coherent thermal features sensed by the infrared camera via PIVlab [40], an approach that has been taken for the purpose of passively sensing mean and turbulent characteristics of the surface flow [41,42]. From our measurement of ship-relative skin velocity, we computed the ship displacement time series: $X(t)=\int_{0}^{t}U_{rel}(t^{\prime })dt^{\prime }$.

For portions of our analysis, it was necessary to consider not only the surface roughness associated with the short scales sensed via PSS (7.51 rad m^−1^ <k< 1000 rad m^−1^) but also the roughness associated with the longer scales sensed via X-band marine radar (0.0159 rad m^−1^ <k< 0.337 rad m^−1^). The total mean square slope (or slope variance) was obtained through integration of the wavenumber slope spectrum obtained via PSS ($S_{pol}(k)$) and the wavenumber slope spectrum inferred via radar ($k^{2}F_{radar}(k)$),


(2.1) $$mss=\int_{k_{min,pol}}^{k_{max,pol}}S_{pol}(k)dk+\int_{k_{min,radar}}^{k_{max,radar}}k^{2}F_{radar}(k)dk.$$

Specifically, the transfer of momentum from wind into surface waves (referred to as the waveform stress in this document) may be inferred through integration of the (polarimeter and radar composite) directional wavenumber slope spectrum S(k,θ) [43],


(2.2) $$\tau_{w}=(0.04\pm 0.02)\rho_{w}u_{*}^{2}\int_{-\pi }^{\pi }\int_{k_{min}}^{k_{max}}k\;{cos}^{2}(\theta )S_{composite}(k,\theta )dkd\theta ,$$

with (0.04±0.02) an empirical coefficient (defining the ranges of variation shown in figure 10*a*,*c*), θ the relative wind-wave direction [43] and [$k_{min}$,$k_{max}$] = [0.0112 rad m^−1^, 1000 rad m^−1^] the domain of wavenumbers resolved by the combination of the polarimetric camera and the marine radar. The value of $u_{*}$ used here was obtained from the COARE parametrization [25], with the shipboard/catamaran scalar measurements as inputs.

#### 2.2.2. Characterization of wave damping

**Figure 5.**
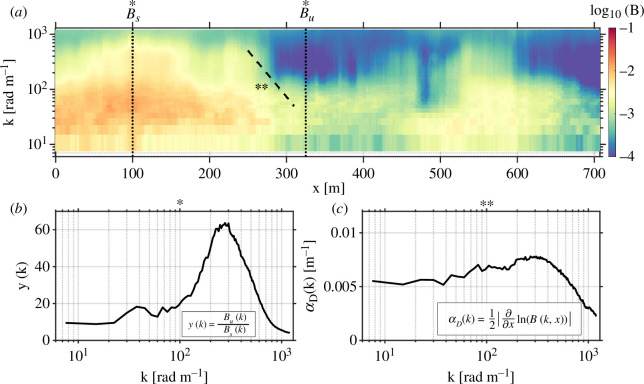
(*a*) The omnidirectional wavenumber saturation spectogram B(x,k). (*b*) The wave damping ratio computed from spectogram slices at dotted lines (*). (*c*) The wave damping coefficient computed from linear fit in semilogarithmic space along dashed line (**).


From our observations of the spatial variation in wavenumber spectrum B(k,x) we computed the damping ratio y(k) and the damping coefficient $\alpha_{D}$. We defined $B_{u}(k)$ as the *unsuppressed* wave spectrum, computed as the average of the wavenumber spectra that constitute the *highest* 10% of mean square slope. Conversely, we defined $B_{s}(k)$ as the *suppressed* wave spectrum, computed as the average of the wavenumber spectra that constitute the *lowest* 10% of mean square slope. The wave damping ratio y(k) (figure 5*b*) is then defined as

(2.3) $$y(k)=\frac{B_{u}(k)}{B_{s}(k)}.$$

Calculation of the damping coefficient required identification of regions of active surface wave suppression (figure 5*a*). To this end, we defined $B_{decay}(k,x)$ as the segment of the spectrogram that decays exponentially from some reference spectral energy density $B_{0}$ with horizontal displacement x. That is,


(2.4) $$B_{decay}(k,x)\equiv B_{0}(k)e^{-2\alpha_{D}x}.$$

The factor of 2 in the argument of the exponent is placed in recognition of the fact that if wave amplitude decays as $\alpha_{D}$, then the wave spectrum (that is, the wave amplitude variance density spectrum) will decay as $2\alpha_{D}$. The damping coefficient was computed in the following manner: for each wavenumber, we applied a 30 s moving window least-squares fit to ln(B(k,x)). We performed an average of $\alpha_{D}$ conditional on the constraints that $R^{2}>$ 0.9 and the estimated $\alpha_{D}>$1⋅10^−3^ m^−1^,


(2.5) $$\begin{array}{ccc} & \alpha_{D}(k)=\frac{1}{N}\sum [\frac{1}{2}|\frac{\partial }{\partial x}\ln(B_{decay}(k,x))|] & \end{array}$$

**Figure 6.**
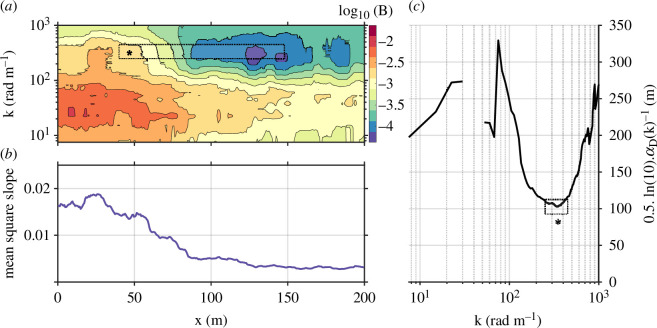
(*a*) Omnidirectional wavenumber saturation spectrogram B(x,k). (*b*) The corresponding spatial variation in mean square slope. (*c*) The distance over which wave spectral energy density falls by one order of magnitude, $D_{\alpha }=0.5\ln(10)\alpha_{D}^{-1}$. The width of the dotted-line box in panel (*c*) (*) indicates the wavenumber range of peak damping. An analogous box is marked in panel (*a*) (*); its height corresponds to the wavenumber range marked in panel (*c*), while the width represents the horizontal spatial scale over which the spectral energy density falls by one order of magnitude.

As shown in figure 6, the calculated value of $\alpha_{D}$ can be re-cast into the form of a characteristic length scale of suppression. In this form, it is consistent with the observed distance over which wave spectral energy density falls by one order of magnitude.

## 3. Results

In our observations, the concentration of surfactant in the SSML was found to be larger for nearshore stations (of the order of 1000 µg l${,}^{-1}$) compared to open ocean stations (of the order of 100 µg l${,}^{-1}$). However, SAS concentration in the SSML of open ocean environments showed a steeper response with increasing SAS concentration in the bulk water [2]. This leads to the general observation that in non-slick regions, the enrichment of organic matter, not the absolute concentration, is higher in open ocean versus coastal environments [2,44,45]. This can be explained by different removal and accumulation processes between these environments, like aggregation processes [46], different solubilities of DOM [44] and photochemical processing of organic matter [45,47]. However, the presence of slicks is understood to skew these measurements, so slick cases are often excluded from such calculations [29]. Including slicks, we find enrichment slightly higher in coastal regions than in the open ocean: $\langle EF_{c}\rangle =$ 2.18, $\langle EF_{o}\rangle =$ 1.29 ($N_{o}=$ 56, $N_{c}=$ 78; difference is statistically significant with p< 0.035). All enrichment factors computed from our observations (including those that did not coincide with wave measurements) are plotted against wind speed in figure 11 of appendix A. In order to quantify the differences observed across other key variables observed in the open ocean and coastal observation sites, two-sample *t*-tests were run across our slate of variables (table 1). Based on these tests, it was determined that the two environments were significantly different in every major category. These differences are underscored by the spread of the distributions of each major variable; this information is presented as distribution percentile values listed in table 3 of appendix C.

**Table 1.** Results from two-sample (open ocean, coastal) *t*-tests run on environmental variables. ‘open ocean’ and ‘coastal’ columns display mean values.

variable	open ocean	coastal	*p*
$U_{10N}$ [m s^−1^]	5.41	3.15	1.77 × 10^−10^
$H_{s}$ [m]	3.53	0.82	7.65 × 10^−39^
$D_{\alpha }$ [m]	194.43	155.11	2.67 × 10^−3^
mean square slope [rad]	4.22 × 10^−2^	1.40 × 10^−2^	5.19 × 10^−21^
$\tau_{w}$ [N m^−2^]	7.38 × 10^−2^	1.18 × 10^−2^	2.00 × 10^−11^
SAS [µg Triton-X eq. l^−1^]	126.52	873.09	1.39 × 10^−2^
CDOM [µg l^−1^]	0.92	1.81	5.05 × 10^−7^
Chl-A [µg l^−1^]	0.16	1.31	1.67 × 10^−4^

**Figure 7.**
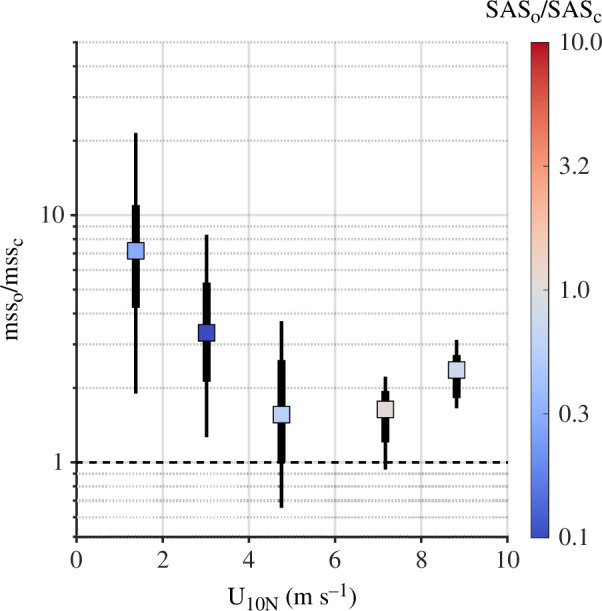
Median ratio of open ocean mean square slope to coastal mean square slope, with thick and thin vertical error bars, respectively, marking the interquartile and interdecile ranges of variation in the mean square slope ratio. The colour indicates ratio of surface active substance concentration.

The substantial difference in surface chemistry between coastal and open ocean waters may help to explain some of the differences observed between coastal [48] and open ocean [31] wave spectra at low to moderate levels of wind forcing. The clear differences in SSML chemistry between coastal and open ocean waters (figure 3*d–f*; table 1) coincided with differences in the observed short-scale wave roughness. This effect is most readily apparent for low wind speeds ($U_{10N}\leq$ 4 m s^−1^), with measurements of mean square slope substantially smaller (2–20 times) in coastal waters than those made in the open ocean, an effect owed in part to the elevated levels (3–10 times) of SAS concentration (figure 7).

**Figure 8.**
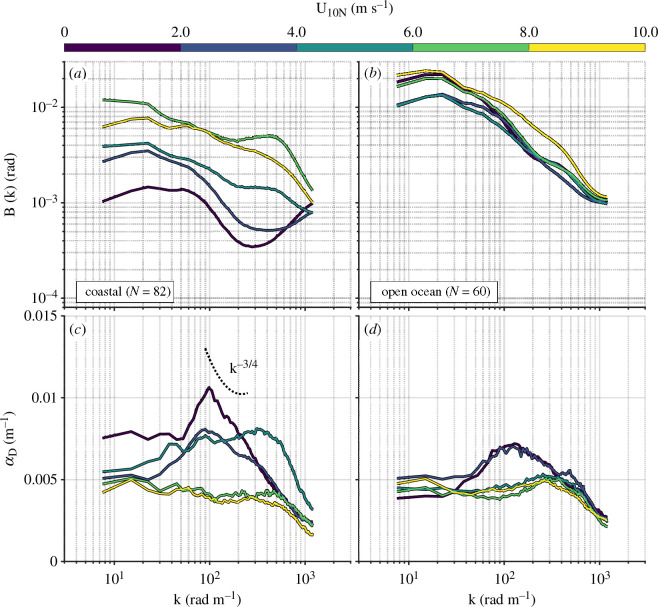
(*a,b*) omnidirectional saturation spectra; (*c,d*) wave damping coefficients. Spectra and damping coefficients were averaged over 2 m s^−1^ bins in $U_{10N}$.

Expanding the view from scale-integrated (mean square slope) to scale-aware (the full wave spectra), we see greater detail in the variation in wave state. We note from figure 8*a* that the slope field noise floor occurs at a wave slope of approximately 0.02 (1.2°). At the lowest wind speeds observed in the coastal region (approx. 0.5 m s^−1^), this limitation prevented measurement of waves with k> 200 rad m^−1^. At higher levels of wind forcing (greater than or equal to 2 m s^−1^), the system was able to reliably observe waves with k up to 1200 rad m^−1^ in almost all cases. Following up to examine regional variation, we find that wave spectra computed from coastal observations occupy a factor of five greater range than those computed from open ocean observations—even when constrained to the same range of wind speeds (figure 8*a*,*b*). Observations of the damping coefficient in the coastal region for $U_{10N}\leq$ 6 m s^−1^ (figure 8*c*) are consistent with key predictions of classic Marangoni–Gibbs wave damping theory. To wit: peak damping occurs for wavenumbers 50 rad m^−1^ <k< 100 rad m^−1^, centimetre-to-decimetre scales for which the dispersion curves for surface transverse and longitudinal waves intersect [11]. Furthermore, post-peak $\alpha_{D}(k)$ falls off as $k^{-3/4}$ for surface gravity waves and curves upwards with increasing wavenumber as capillarity becomes the dominant restoring force; the dotted curve in figure 8*c* indicates the damping coefficient predicted from theory [10,49], with an arbitrary vertical offset. As this curve was produced with an assumed clean water value of surface tension (σ= 72 mN m^−1^), the extension of $k^{-3/4}$ behaviour for higher wavenumber at the lowest level of wind force is probably the result of reduced surface tension—and therefore dominance of gravity as a restoring force for centimetre-scale waves. The continuation of $k^{-3/4}$ to k≈ 200 rad m^−1^ suggests a reduced surface tension σ≈ 60 mN m^−1^; this is consistent with laboratory measurements of surface tension in water with a similar concentration (approx. 1000 µg l^−1^) of Triton X−100 [7].

The breadth of the distinction between open ocean and coastal conditions makes challenging the identification of a single variable as the dominant predictor for the suppression of surface waves. We subjected our observational data to partial least squares (PLS) regression. For this process, the waveform stress was set as the ‘response’ variable, while all others were set as ‘predictor’ variables. The waveform stress was chosen as the response variable in order to explicitly connect the suppression of surface waves to the exchange of energy across the air–sea interface. The variable importance in projection (VIP) [50] scores are listed in table 2, with VIP greater than 1 (a traditional cut-off for particular importance) marked with bold text.

**Table 2.** Variable importance in projection (VIP) from partial least squares (PLS) calculation for all observations. Predictors in left column were considered with respect to the response variable of the waveform stress ($\tau_{w}$). Variables with VIP scores >1 are marked with red text.

predictor	VIP
$U_{10N}$[m s^−1^]	**1.86**
$H_{s}$[m]	0.92
$D_{\alpha }$[m]	**1.04**
SAS [g Triton-X eq. l^−1^]	0.64
CDOM [µg l^−1^]	0.23
Chl-A [µg l^−1^]	0.37

## 4. Discussion

**Figure 9.**
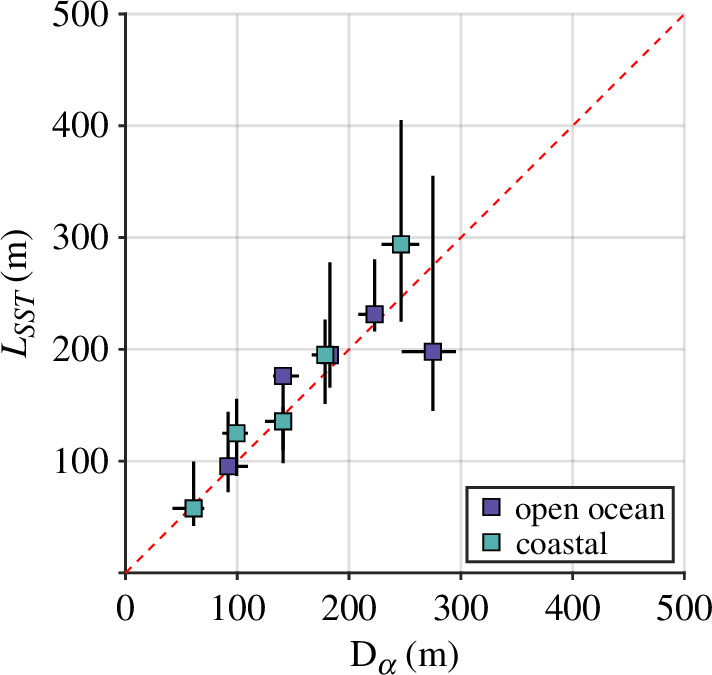
Scatterplot comparing variation of peak wave damping length scale $D_{\alpha }$ and the peak length scale obtained from the SST wavenumber spectrum. Markers represent the median position of data in each of five evenly sized bins, while black bars indicate the interquartile range of variation within each bin.

Regional variability in surface slicks (i.e. in their concentration and spatial arrangement) bears greatly on the wave field. By examining the behaviour of $\alpha_{D}(k)$ averaged by wind speed and separated by region, we see that two well-defined clusters emerge: (i) cases for which $U_{10N}\leq$ 6 m s^−1^ and (ii) all other cases. Over the short gravity wave regime (decimetre- to centimetre-scale wavelengths), this low-wind coastal cluster exhibits damping coefficients, which are 2–4 times higher than those in the other cluster. This delineation at $U_{10N}=$ 6 m s^−1^ is consistent with past observations of surface slicks in the warm coastal ocean [51]. We find significant differences between the concentrations of SAS, CDOM and Chl-A measured in open ocean and coastal waters (table 1). However, the PLS analysis does not indicate that mean concentration of any of these variables is a strong driver of $\tau_{w}$ (table 2). Although the difference between coastal and open ocean concentrations of these substances is statistically significant, that appears to be driven in large part by the disparity at the high end of the distribution; e.g. the 90th percentile of table 3. Furthermore, we lose a great deal of context by bin-averaging measures of damping, which are themselves averaged in space and time. The suppression of surface waves by slicks is highly localized, yet we report mean parameters obtained over individual approximately 20 min/1 km acquisition periods. This averaging obscures the effect of spatial variability in features that are characteristically streaky and filamentous [18,52]. By recasting the damping ratio ($\alpha_{D}$) in terms of a characteristic length scale of wave damping ($D_{\alpha }$) we can recover some of the spatial information lost during the averaging process. We see that for low to moderate wind speed in coastal regions, wave suppression was strong and localized to thin regions of damping ($D_{\alpha }<$ 100 m; figure 2). However, our technique for determining the damping coefficient (and therefore the length scale $D_{\alpha }$) is agnostic to the presence of slicks; we simply identified periods of exponential decay or growth of the wave field. In order to provide an independent quantification of the spatial scale of persistent (non-wave) features on the sea surface, we subjected our infrared SST measurement to the same spatial projection used on the short-wave spectra, yielding the spatial variation of SST. We extracted the length scale corresponding to the spectral peak from each acquisition’s spectrum in order to produce $L_{SST}$, the length of peak variation in SST. A scatterplot of $D_{\alpha }$ and $L_{SST}$ is provided in figure 9. This result provides strong evidence that the damping spatial scale $D_{\alpha }$ is intimately connected to the spatial scale of the slick features themselves.

**Figure 10.**
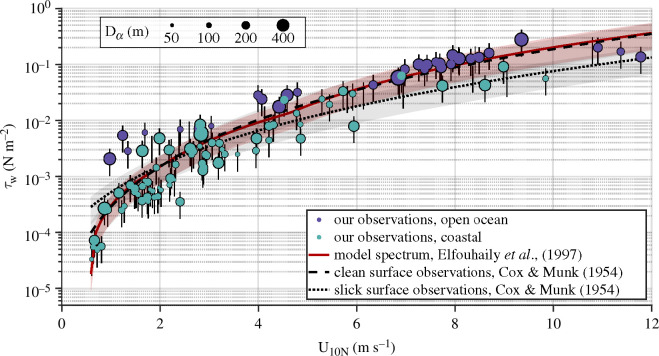
Variation of waveform stress with wind speed, with size indicating $D_{\alpha }$ (figure 6). Violet circles mark open ocean measurements and teal circles mark coastal measurements, while the red curve was derived from Elfouhaily *et al*.’s parametrized wavenumber spectrum [53] and the black curves were derived from Cox & Munk’s classic field measurements of surface slope variance over clean (dashed line) and slick (dotted line) sea surfaces [54].

**Figure 11.**
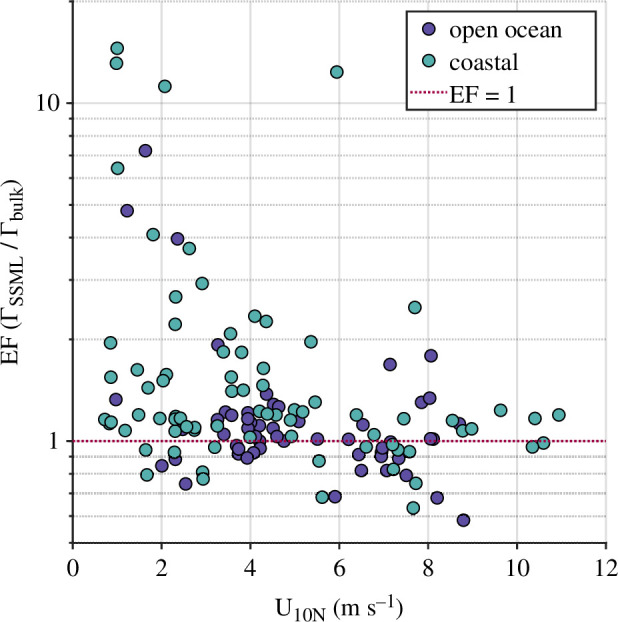
Variation of the enrichment factor EF = $\Gamma_{SSML}/\Gamma_{bulk}$ (the ratio between SSML and bulk concentrations of SAS) as a function of wind speed ($U_{10N}$). Horizontal dotted red line indicates a ratio of 1 (concentrations in SSML and bulk water are equal).

Suppression of surface waves by biogenic slicks is interesting enough in its own right. However, it is the intimate relationship between short surface waves and the mediation of air–sea fluxes [43] that provides the strongest motivator for continued study in this area. The effect of surface slicks to substantially reduce wave slope variance (particularly at wavelength scales of centimetres to decimetres) is, therefore, expected to result in lower momentum imparted by wind to the surface wave field. Our observations (as communicated in figure 10) bear this out: cases for which surface waves are strongly damped over short spatial distances ($D_{\alpha }$ of order 10 m) are associated with a substantial reduction in waveform stress. The cluster of observations made in coastal regions with low to moderate wind speed is further divided by damping strength, with $\tau_{w}$ varying by a factor of 2–10 with $D_{\alpha }$ for a given wind speed. We note that the exceptional agreement between the clean surface observations of Cox & Munk [54] and the model spectrum output of Elfouhaily *et al*. [53] is a matter of course: Elfouhaily *et al*.’s model was tuned to reproduce Cox & Munk. However, we have included the model spectrum results as they were generated over a wavenumber range approximately equal to the one resolved by our field observations and remote sensing techniques (1 × 10^−3^ rad m^−1^ ≤k≤ 1 × 10^3^ rad m^−1^).

To draw an analogy, streaks and filaments of surfactant act like a brake on the wave field. If this brake is applied with sufficient strength, wind wave growth is stunted and the sea is not permitted to fully develop. This stifling of wave field development reduces the exchange of momentum, heat and gas between atmosphere and ocean in the region of the slick. Knowledge of mean environmental conditions (e.g. wind speed, long-wave state, chemical concentration; table 1) serves as a useful heuristic; however, this knowledge alone is insufficient to describe the wave suppression observed (particularly in coastal waters). We find that multiple environmental factors contribute to the suppression of short-scale surface waves by biogenic slicks—prominent among them, the strength of wave damping within the localized slick regions (table 2). The effort (before, during and after field observations are complete) and technology required to make this measurement are considerable. However, it may be the case that remote sensing techniques with high spatial resolution and sufficiently wide adoption (e.g. marine *x*-band radar, figure 1*a*) offer a means of quickly determining the spatial damping characteristics of biogenic slicks.

## 5. Conclusions

We report on field observations of the damping of short (metre- to centimetre-scale) ocean surface waves in the presence of biogenic slicks. These observations were made in the open ocean waters of the Western Equatorial Pacific and the organic material-rich coastal waters of the East Timor Sea. By acquiring short-wave slope fields at high spatial and temporal resolution during slow ship transects through slicks, we were able to compute the absolute ratio of damped to undamped sea states (i.e. the damping ratio) and the spatial decay rate of waves on the margins of slicks (i.e. the damping coefficient). In tandem with the wave observations, the chemical and biological characteristics of the sea surface microlayer and underlying surface water were measured via a minimally invasive, remotely operated catamaran. We observed significant differences in mean conditions between open ocean and coastal environments: coastal waters were characterized by lower wind forcing, less energetic surface gravity wave fields and higher concentrations of CDOM, Chl-A and SAS. Our measurements of the spectral wave damping coefficient showed evidence of Marangoni–Gibbs damping in the surface gravity wave regime, with the waves most intensely suppressed falling in the range of 5–15 centimetres in wavelength. We reinterpreted the damping coefficient to provide a characteristic length scale of wave suppression, $D_{\alpha }$: the distance over which wave amplitude falls by an order of magnitude. This representation allows us to consider the spatial features of a diverse and heterogeneous region of slicks within a single integral quantity. For cases in which wind forcing is low to moderate over coastal regions, it is the strength of surface wave damping (or, the narrowness of the suppression region $D_{\alpha }$) which most clearly delineates instances of nominal and diminished waveform stress. This reduction of momentum input to the wave field has a cascading effect on all manner of processes, modifying wind/Stokes drift currents and air–sea fluxes of heat and gas. The mere presence of surfactant impacts the surface wave field and air–sea fluxes in a way that is usually not quantified (or even parametrized) in physical descriptions of air–sea interaction. Our results indicate that the spatial arrangement and density of biochemical ocean surface slicks have the potential to magnify the effect of this variability, with innumerable surface roughness and temperature fields possible for any particular mean concentration of surfactant. Given the ubiquity of slicks on the surface of the coastal ocean, we infer that this effect is a strong (and possibly dominant) contributor to the difficulty of modelling and parametrizing wave growth and air–sea interaction in nearshore zones.

## Figures and Tables

**Figure 12.**
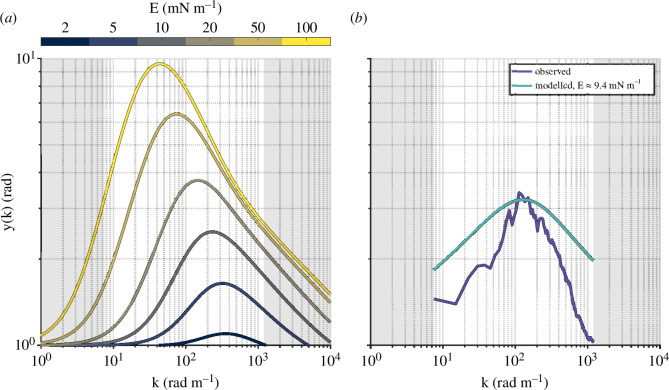
(*a*) Variation of the wave damping ratio with modelled [10] surface elasticity, setting phase angle $\theta =0^{\circ }$. (*b*) An example of an observed damping ratio (violet) overlaid with a modelled damping ratio (teal) produced via least-squares fit around the spectral peak; this fit yields an estimate of the surface elasticity *E* [21]. The light (non-grey) region demarcates the spectral domain of the field observations.

## Data Availability

The surface chemistry data collected via S^3^ catamaran are publicly available through PANGAEA [55]. Relevant data and codes used in the preparation of this manuscript are publicly available through the Columbia Academic Commons [56].

## Appendix A. Sea surface microlayer chemistry

We show the variation of SAS enrichment factor with wind speed in figure 11. Since we include cases in which visible slicks were present, enrichment tended to be higher in coastal regions than in the open ocean.

## Appendix B. Inference of surface rheology

Given observations of the damping ratio y(k), one may infer the value of the elasticity of the surface sea water [10,21,57]. The damping ratio may be modelled as a function of wavenumber k and radian frequency ω, with parametric dependence on surface viscoelastic properties,

(B 1)
$$\begin{array}{ccc} & y(\omega ,k)=\frac{1+X[\cos(\theta )-\sin(\theta )]+XY-Y\sin(\theta )}{1+2X[\cos(\theta )-\sin(\theta )]+2X^{2}} & \end{array}$$

with

$$\begin{array}{rl} X & =\frac{Ek^{2}}{\sqrt{2\mu \omega^{3}}}\\ Y & =\frac{Ek}{4\mu \omega } \end{array}.$$

The complex elasticity is provided in the form of E (elastic modulus) in N m^−1^ and θ (phase angle between maximum interfacial compression and minimum surface tension) in radians; μ is the dynamic viscosity in N s m^−2^.

For each observational case, we computed a family of modelled damping ratios [10] corresponding to a range of elastic moduli (figure 12*a*). We then calculated a least-squares best fit around the peak of the observed damping ratio in order to estimate the elastic modulus for that particular case (figure 12*b*). Following [10], we fixed surface tension σ= 0.072 N m^−1^. For σ> 0.040 N m^−1^, this constitutes less than 10% error in ω over the centimetre- and larger-scale waves for which significant damping was observed.

## Appendix C. Quantile ranges of key environmental variables

See table 3.

**Table 3.** Quantile values of environmental variables observed in open ocean (bold) and coastal (italic) cases. The 50th percentile is the median; the 25th–75th percentiles serve as bounds to the interquartile range, while the 10th–90th percentiles serve as bounds to the interdecile range.

variable	10th	25th	50th	75th	90th
$U_{10N}$ [m s^−1^]	1.6, 1.7	3.1, 1.8	4.5, 2.5	7.7, 4.0	8.7, 5.9
$H_{s}$ [m]	0.60, 0.44	2.42, 0.50	3.85, 0.59	4.56, 1.16	5.36, 1.42
$D_{\alpha }$ [m]	91.9, 64.5	135.4, 91.4	185.9, 141.0	229.12, 195.7	295.6, 262.6
$L_{SST}$ [m]	90.6, 55.3	144.0, 99.6	199.0, 151.2	298.4, 259.3	415.7, 407.2
$10^{3}\cdot$ mss [rad]	25.3, 3.4	29.8, 5.0	41.4, 9.0	50.3, 21.8	55.4, 29.7
$10^{3}\cdot \tau_{w}$ [N m^−2^]	2.9, 0.2	7.9, 0.6	54.1, 2.5	126.5, 7.9	145.5, 32.7
SAS [µg TX eq. l^−1^]	64.0, 74.0	66.3, 80.0	76.7, 114.5	91.0, 164.3	313.3, 2390.3
CDOM [µg l^−1^]	0.40, 0.49	0.62, 1.04	0.82, 1.35	1.15, 1.93	1.53, 4.00
Chl-A [µg l^−1^]	0.11, 0.12	0.13, 0.14	0.14, 0.20	0.20, 0.25	0.22, 4.21
